# Electrochemical Biosensor Based on Horseradish Peroxidase and Black Phosphorene Quantum Dot Modified Electrode

**DOI:** 10.3390/molecules28166151

**Published:** 2023-08-21

**Authors:** Xiaoqing Li, Fan Shi, Lisi Wang, Siyue Zhang, Lijun Yan, Xiaoping Zhang, Wei Sun

**Affiliations:** 1 Key Laboratory of Functional Materials and Photoelectrochemistry of Haikou, Key Laboratory of Laser Technology and Optoelectronic Functional Materials of Hainan Province, College of Chemistry and Chemical Engineering, Hainan Normal University, Haikou 571158, China; l1x2q3_li@126.com (X.L.); shifan1218@126.com (F.S.); wanglisis@126.com (L.W.); bjyxszd_85105@163.com (S.Z.); ylj0103@163.com (L.Y.); 070911@hainnu.edu.cn (X.Z.); 2 College of Health Sciences, Shandong University of Traditional Chinese Medicine, Jinan 250355, China

**Keywords:** black phosphorene quantum dots, horseradish peroxidase, direct electrochemistry, electrochemical detection

## Abstract

Black phosphorene quantum dots (BPQDs) were prepared by ultrasonic-assisted liquid-phase exfoliation and centrifugation with morphologies proved by TEM results. Furthermore, an electrochemical enzyme sensor was prepared by co-modification of BPQDs with horseradish peroxidase (HRP) on the surface of a carbon ionic liquid electrode (CILE) for the first time. The direct electrochemical behavior of HRP was studied with a pair of well-shaped voltammetric peaks that appeared, indicating that the existence of BPQDs was beneficial to accelerate the electron transfer rate between HRP and the electrode surface. This was due to the excellent properties of BPQDs, such as small particle size, high interfacial reaction activity, fast conductivity, and good biocompatibility. The presence of BPQDs on the electrode surface provided a fast channel for direct electron transfer of HRP. Therefore, the constructed electrochemical HRP biosensor was firstly used to investigate the electrocatalytic behavior of trichloroacetic acid (TCA) and potassium bromate (KBrO_3_), and the wide linear detection ranges of TCA and KBrO_3_ were 4.0–600.0 mmol/L and 2.0–57.0 mmol/L, respectively. The modified electrode was applied to the actual samples detection with satisfactory results.

## 1. Introduction

As a rising star of a new two-dimensional layered-structure material, black phosphorene (BP) has the advantages of low defect density, high specific surface area, adjustable particle size, fast hole mobility, long carrier lifetime, good biocompatibility, and direct band gap [1,2]. Due to the good electrical and optical properties, such as excellent interface activity, high photoelectric properties, fast electron transfer rate, and good conductivity, BP and related composites are widely used in solar cells, electronic devices, transistors, and other fields [3,4,5]. However, BP is unstable in oxygen-containing aqueous solution, which limits its application fields, and thus, it is a great challenge to the applications of BP and BP-based composites [6,7,8,9]. In general, BP can be easily obtained by liquid phase ultrasonic exfoliation under strict oxygen-free and water-free conditions with the protection of organic solvents [10,11]. Brent et al. prepared 1–5 layers of BP by the liquid phase stripping method with N-methyl-2-pyrrolidone as the solvent [12]. Different methods, including the surface hybridization, doping, and functionalization of BP, can reduce the active reaction of the P atom with O_2_ or H_2_O, and solve the easy oxidization of BP [13,14]. Li et al. fabricated a poly(3,4-ethylenedioxythiophene)-poly(styrenesulfonate) and BP (PEDOT: PSS-BP) composite membrane electrode for electrochemical applications [15]. Liu et al. coated polymethyl methacrylate on the surface of BP to isolate air and prevent oxidation [16]. Generally speaking, the selected solvent should have surface energy similar to that of BP and then effectively protect BP from oxidation [17].

Black phosphorene quantum dots (BPQDs) are one form of nanostructured BP with unique optical properties and good biocompatibility, which has shown better performances in biomedical fields, such as drug delivery, cell tracking, and so on [18,19,20]. But, there are few reports on the applications of BPQDs in electrochemical sensors before 2017 [21]. Recently, infrequent reports using BPQDs have been published. Ding et al. prepared a BPQDs doped nano-ZnO composite for detection of hydrogen peroxide [22]. Zhang et al. synthesized BPQDs from bulk BP by the liquid stripping method with an average particle size of 8.2 nm, which could significantly catalyze the oxidation process of Ru(bpy)_3_^2+^ and could be used as a co-reactant in an electrochemiluminescence system for sensitive detection of dopamine [23]. However, no documents about the applications of BPQDs in electrochemical enzyme sensors have been reported. Since BPQDs exhibit excellent electrochemical properties, the direct modification of BPQDs on the electrode for the construction of an electrochemical enzyme biosensor is worth investigating to extend the applications of BPQDs-based electrochemical biosensors.

As a class of environmental contaminant, trichloroacetic acid (TCA) exhibits certain carcinogenic effects and can cause a serious impact on human life [24]. It can be found in not only industrial wastes, but also drinking water as a result of disinfection by chlorine [25]. Various analytical methods have been designed for TCA analysis, such as gas chromatography [26], high-performance liquid chromatography [27], capillary electrophoresis [28], and mass spectrometry [29]. As a widely used food additive and a water disinfection byproduct, bromate is considered to be potentially carcinogenic and nephrotoxic, and has been identified as a category I group B2 carcinogen [30]. The acceptable maximum levels of TCA and bromate in drinking water set by China National Standards are 0.1 mg/L and 10 µg/L, respectively [31]. Also, the abuse of food additives containing potassium bromate is hazardous to human health. Therefore, it is essential to develop a rapid, reliable, and sensitive method for the detection of TCA or bromate.

In this paper, BPQDs were prepared by ultrasonic-assisted liquid-phase exfoliation and centrifugation using multilayer black phosphorus nanoplates (BPNPs) dispersed in 1-methyl-2-pyrrolidinone (NMP). Then, a BPQDs-decorated carbon ionic liquid electrode (CILE), which was used as the base electrode for horseradish peroxidase (HRP) immobilization, was prepared by simple drop-casting. CILE is made up of carbon paste and ionic liquid, which have been proven to exhibit many advantages, including wide electrochemical windows, good antifouling ability, high conductivity, and inherent electrocatalytic ability, and which are selected as the basic electrode for the modification due to the excellent electrochemical performances [32,33,34]. The BPQDs provided a large surface area for loading more HRP molecules with high conductivity and good biocompatibility. The fabricated modified electrode (Nafion/HRP/BPQDs/CILE) had good electrochemical and electrocatalytic performances, which was applied to the detection of real samples with satisfactory results.

## 2. Results and Discussion

### 2.1. Characterizations

**Figure 1:**
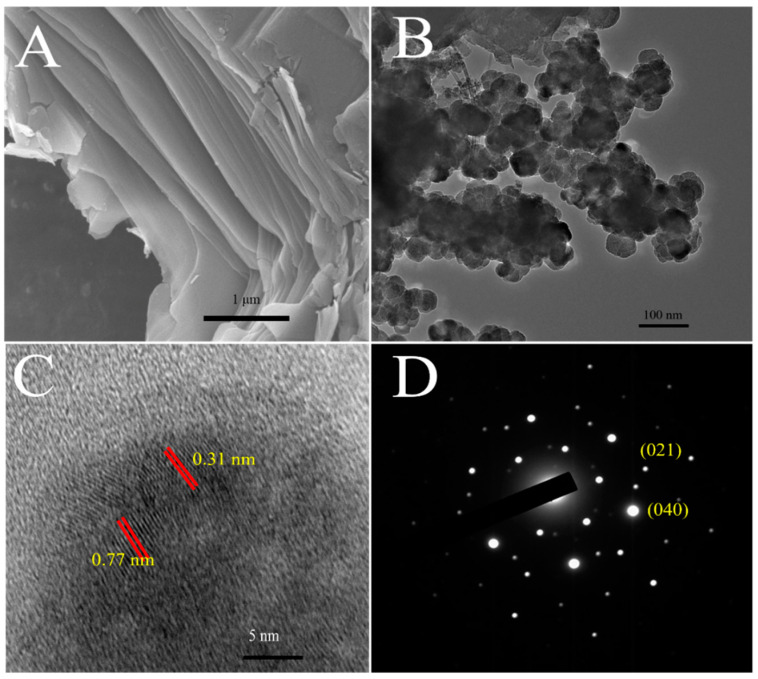
(**A**) SEM image of BPNPs; (**B**) TEM image of BPQDs; (**C**) HRTEM image of BPQDs with different lattice fringes; (**D**) selected area electron diffraction pattern of BPQDs.

The BPNPs used were characterized by SEM, with the result shown in Figure 1A, which presented a typical multilayered flake structure. BPQDs were prepared according to the previous report [22], and the morphology was characterized by TEM. As shown in Figure 1B, a tiny and uniform flake-like morphology appeared, with the average diameter of 10 nm, which showed obvious differences to that of BPNPs. The HRTEM was further recorded and is shown in Figure 1C; the lattice fringes could be ascribed to the (040) and (021) planes of BPQDs crystal. From Figure 1D, it could be seen the typical selected area electron diffraction pattern of BPQDs, which demonstrated the crystallinity of the structure.

**Figure 2:**
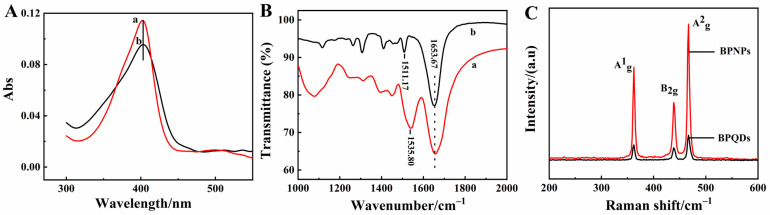
(**A**) UV-Vis absorption spectra of 5.0 mg/mL HRP in water (curve a) and 1.0 mg/mL BPQDs solution (curve b); (**B**) FT-IR spectra of HRP (curve a) and BPQDs-HRP composite (curve b); (**C**) Raman spectra of BPNPs and BPQDs.

UV-Vis absorption spectroscopy is a common spectral method used to detect whether the original biological structure of the enzyme is denatured [35]. As shown in Figure 2A, the absorption Soret band of HRP in water (curve a) had the same position in the BPQDs solution (curve b) at 402.6 nm, indicating that the native structure of HRP was not changed with BPQDs. The structure information of the HRP molecule was further investigated by FT-IR using a KBr disk, with results shown in Figure 2B. The infrared absorption bands of amide I and amide II in the HRP structure were 1653.67 cm^−1^ and 1535.80 cm^−1^ (curve a), respectively. The bands of amide I and amide II of HRP in BPQDs-HRP composites were 1653.67 cm^−1^ and 1511.17 cm^−1^ (curve b), respectively. The position of the infrared absorption band was almost the same, with a slight shift, which revealed the certain interaction between HRP and BPQDs. It indicated that HRP maintained a good conformation in the composite and did not change after HRP mixing into the BPQDs solution, which could be attributed to the excellent biocompatibility of BPQDs.

Furthermore, BPQDs were characterized by Raman spectroscopy, with the results shown in Figure 2C. Three typical peaks appeared at 361.1 cm^−1^, 440.4 cm^−1^, and 466.6 cm^−1^, respectively, which were corresponding to the one out-of-plane vibration mode A^1^_g_ and two in-plane vibration modes B_2g_ and A^2^_g_ of BPQDs, and the results were close to other literature [36,37]. According to the reference [38], the intensities of the A^1^_g_, B_2g_, and A^2^_g_ modes increased with the number of layers, and the intensity ratio (A^1^_g_/A^2^_g_) values of BPNPs and BPQDs were 0.70 and 0.62, respectively. A^1^_g_/A^2^_g_ ratio values were larger than 0.6, demonstrating the evidence for lower oxidation of BPQDs [39]. In comparison with BPNPs, the Raman peaks displayed lightly blue shifts of BPQDs, which could stem from the weakened interlayer Van der Waals force as a result of reduced thickness [40].

**Figure 3:**
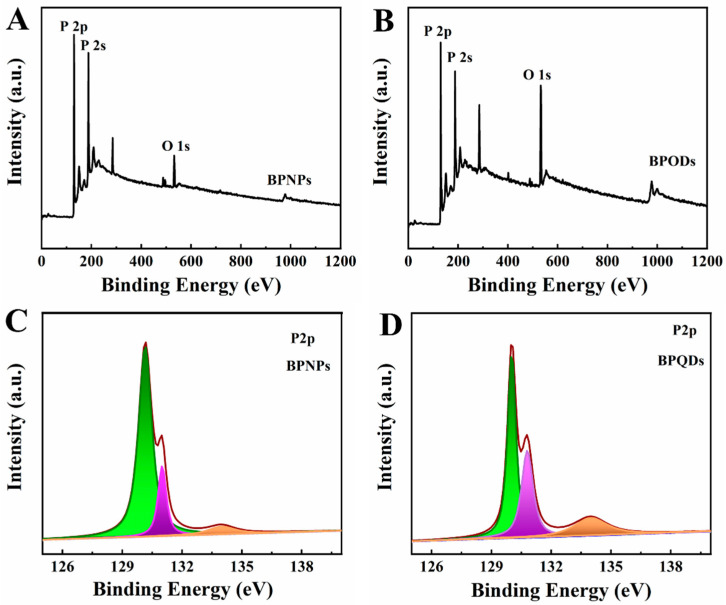
XPS spectra of (**A**) BPNPs and (**B**) BPQDs and P 2p spectra of (**C**) BPNPs and (**D**) BPQDs.

XPS is used to evaluate the structural and chemical states of the elements of BPNPs and BPQDs, with the result shown in Figure 3. The survey spectrum of BPNPs (Figure 3A) and BPQDs (Figure 3B) illustrated the presence of P and O elements. High-resolution XPS spectra of the P 2p signal enable a precise investigation of the oxidation state of P. In the P 2p spectra of BPNPs (Figure 3C), there were two peaks at 130.2 eV and 131.0 eV, corresponding to single spin-orbit P–P 2p^3/2^ (green) and P–P 2p^1/2^ (purple), respectively [41]. The third relatively weak peak at 134.0 eV was observed by slight oxidation of BPNPs, which was attributed to P–O bonds (brown) [42]. In the spectrum of BPQDs (Figure 3D), the intensity of P–O bonding of BPQDs was higher than of BPNPs, indicating that the electron of P was taken by O in the process of exfoliated BPQDs. This suggests that the exfoliated nanosheets are more prone to being oxidized.

**Figure 4:**
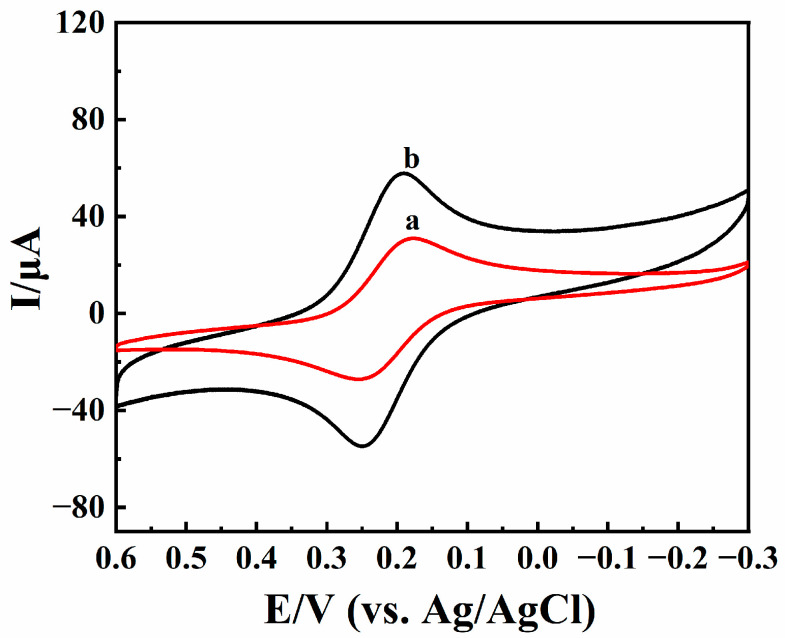
Cyclic voltammograms of CILE (curve a) and BPQDs/CILE (curve b) with the scan rate of 100 mV/s in 1.0 mmol/L K_3_[Fe(CN)_6_] and 0.5 mol/L KCl solution.

Cyclic voltammetric curves of BPQDs/CILE and CILE were checked by using 1.0 mmol/L K_3_[Fe(CN)_6_] solution as an electrochemical probe, with curves shown in Figure 4. A pair of well-shaped redox peaks appeared on the bare CILE (curve a). The redox peak currents on BPQDs/CILE (curve b) were significantly higher than those of bare CILE, with the oxidation and reduction peak currents increased for 1.62 and 1.69 times after the double-large charging current were substrated. Furthermore, the effective electrochemical surface area of BPQDs/CILE was calculated according to the Randles–Sevcik equation [43], (Ip = 2.69 × 10^5^AD^1/2^n^3/2^υ^1/2^C). The effective area of BPQDs/CILE was calculated to be 0.204 cm^2^, which was 1.63 times larger than that of bare CILE (0.126 cm^2^). The results showed that the existence of BPQDs greatly increased the effective surface area of the working electrode, which was beneficial to providing a large electrode interface and improving the electrochemical performance.

### 2.2. Direct Electrochemistry

**Figure 5:**
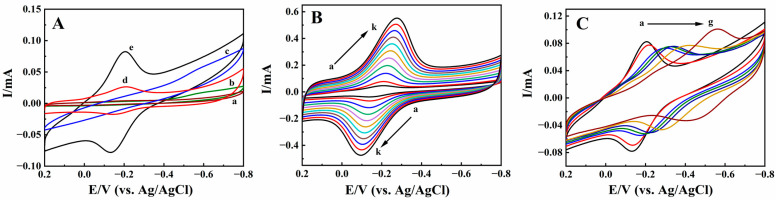
(**A**) Cyclic voltammograms of (a) Nafion/CILE, (b) CILE, (c) Nafion/BPQDs/CILE, (d) Nafion/HRP/CILE, and (e) Nafion/HRP/BPQDs/CILE in pH 2.0 PB with a scan rate of 100 mV/s; (**B**) Cyclic voltammograms of Nafion/HRP/BPQDs/CILE with different scan rates in pH 2.0 PB (from a to k: 50, 100, 200, 300, 400, 500, 600, 700, 800, 900, 1000 mV/s); (**C**) Cyclic voltammograms of Nafion/HRP/BPQDs/CILE at different pH PB (from a to g, representing pH values of 2.0, 3.0, 4.0, 5.0, 6.0, 7.0, 8.0) with a scan rate of 100 mV/s.

Cyclic voltammetry was used to investigate the properties of different modified electrodes, with curves shown in Figure 5A. The largest redox peaks current appeared in pH 2.0 PB; therefore, it was selected as the optimum pH to study the electrochemical behaviors. In pH 2.0 PB, on Nafion/CILE (curve a), CILE (curve b), and Nafion/BPQDs/CILE (curve c), no redox peaks appeared, indicating that there was no electrochemical reaction at the selected potential windows. When HRP was embedded, a couple of well-defined redox peaks appeared on Nafion/HRP/BPQDs/CILE (curve e), with the peak currents as 38.90 μA (Ipc) and 47.92 μA (Ipa), respectively. The cathodic (Epc) and anodic (Epa) peak potential were located at −0.205 V and −0.132 V with the peak-to-peak separation (ΔEp) as 73 mV, which could be considered as a quasi-reversible reaction of the HRP Fe(III)/Fe(II) redox active center. The equilibrium peak potential (${E^{0}}^{\prime }$), which was calculated from the equation as ${E^{0}}^{\prime }$ = (Epa + Epc)/2, was estimated as −0.169 V. Compared with Nafion/HRP/BPQDs/CILE, a pair of redox peaks also appeared on Nafion/HRP/CILE (curve d), with the peak currents as 14.16 μA (Ipc) and 9.50 μA (Ipa), respectively. The ratios of the cathodic and anodic peak currents of two HRP modified electrodes (curve e and d) were 2.75 and 5.04, respectively, which was larger than the increase in the effective surface area of the BPQDs modified CILE (1.63). Therefore, the presences of BPQDs on the electrode surface plays key roles in promoting the electron transfer rate of HRP Fe(III)/Fe(II) [44]. BPQDs have advantages including large specific surface areas and good electrical conductivity and centers. When BPQDs are present on the CILE surface, they can provide more electron transfer paths for HRP Fe(III)/Fe(II) to exchange electrons with CILE.

The effect of scan rate (υ) on the cyclic voltammetric responses of Nafion/HRP/BPQDs/CILE was studied from 50 to 1000 mV/s, and the curves are shown in Figure 5B. The cathodic and anodic peak currents increased simultaneously with the increase in scan rate. The redox peak potentials also shifted slightly with the ΔEp value enlarged, indicating a quasi-reversible process. The linear regression equations of the current and scan rate were computed as Ipc (mA) = 29.25 υ (V/s) +0.7901 (γ = 0.992) and Ipa (mA) = −25.69 υ (V/s) −2.267, (γ = 0.991), respectively. Also, the linear relationships between redox potential (Ep) and lnυ were obtained as Epc (V) = −0.0521 lnυ (V/s) −0.2752 (γ = 0.993) and Epa (V) = 0.0523 lnυ (V/s) −0.0845 (γ = 0.992), respectively. According to Laviron’s model [45,46], the corresponding electrochemical parameters were calculated with the electron transfer coefficient (α), the values of the electron transfer number (n), and the apparent electron transfer rate constant (*k_s_*) as 0.50, 0.986, and 1.54 s^−1^, respectively. The *k_s_* value is larger than that of HRP on Nafion/HRP/AuNTs/CILE (1.01 s^−1^) [47], Nafion/HRP/Co_3_O_4_/CILE (0.94 s^−1^) [48], and HRP/nano-Ni-SnO_2_/GCE (1.10 s^−1^) [49], indicating that the existence of BPQDs played an important role for the fast electron transfer between HRP and the electrode surface. The average surface concentration (Γ*) of electroactive HRP on the modified electrode was calculated based on the equation Q = nFAΓ* [50]. The value of Γ* was estimated to be 3.54×10^−9^ mol/cm^2^, and the total amount of HRP on the modified electrode interface was 2.98×10^−8^ mol/cm^2^, which accounted for 11.88% of HRP molecules on the electrode surface taking part in the electrode reaction.

The pH of buffer solution could affect the electrochemical behavior of HRP on the modified electrodes, and the results are shown in Figure 5C. From the pH range from 2.0 to 8.0, the ${E^{0}}^{\prime }$ value shifted negatively with the increase in pH. It can be seen that the largest redox peaks current appeared in PB 2.0 (curve a). The electrochemical reaction involves the proton, and Nafion film can protect the HRP molecules on the electrode surface; therefore, pH 2.0 PB was used in this experiment, which could provide enough protons for the electrode reactions. There was a good linear relationship between ${E^{0}}^{\prime }$ and the different pH with the linear regression equation as ${E^{0}}^{\prime }$ (V) = −0.0545 pH −0.0431 (γ = 0.991). The slope value was −54.5 mV/pH, which was slightly smaller than the theoretical value of −59.0 mV/pH for the same amounts of protons and electrons [51], indicating that microenvironment changes could affect the electrochemical behavior of HRP.

### 2.3. Electrocatalytic Performances of TCA and KBrO_3_

Due to the excellent catalytic activity of HRP on the modified electrode, the electrocatalytic performances of Nafion/HRP/BPQDs/CILE for two halide compounds were studied. These compounds are stable in aqueous solution and cannot be degraded on the commonly used electrode system. The presence of HRP on the modified electrode can greatly decrease the overpotential of the analytes on the electrode due to its catalytic effects.

**Figure 6:**
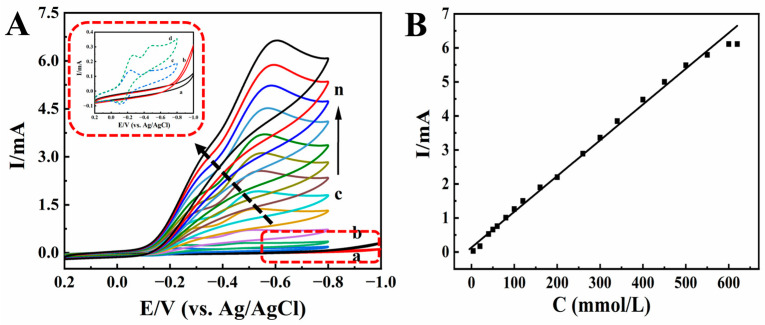
(**A**) Cyclic voltammograms of Nafion/BPQDs/CILE in 0.1 mol/L pH 2.0 PB with (a) 0.0, (b) 5.0 mmol/L TCA, and Nafion/HRP/BPQDs/CILE with 4.0, 20.0, 40.0, 80.0, 120.0, 160.0, 200.0, 260.0, 340.0, 400.0, 500.0, 600.0 mmol/L TCA (curves c–n) at the scan rate of 100 mV/s. Inset was magnification of curves from a to d. (**B**) Linear relationship of catalytic reduction peak currents and TCA concentration.

As an organic halide, TCA is widely used for herbicides and preservatives. Therefore, it is important to find a sensitive method for TCA analysis [52]. The redox protein-modified electrodes exhibit excellent electrocatalytic activity to TCA [53]. Figure 6A shows the electrocatalytic behaviors of modified electrodes for TCA. When different concentrations of TCA were gradually added to pH 2.0 PB, the reduction peak current increased gradually, while the oxidation peak current decreased gradually until it disappeared (curves c-n). With the increase in TCA concentration, there was a good linear relationship between the catalytic reduction peak current and the TCA concentration in the range of 4.0 to 600.0 mmol/L (Figure 6B). The linear regression equation was Ip (mA) = 0.0105 C (mmol/L) + 0.1335 (γ = 0.994), and the detection limit was 0.03 mmol/L (3S/N), where S is the standard deviation of the blank, and N is the slope of the linear calibration curve. When the concentration of TCA exceeded 600.0 mmol/L, the reduction peak current remained basically unchanged, showing a typical Michaelis–Menten kinetics mechanism. In order to confirm HRP catalytic behavior, comparison experiments were explored with Nafion/BPQDs/CILE as the working electrode, which showed no redox peaks appeared for TCA (curves a, b, and inset of Figure 6A), indicating that HRP played key roles in the electrocatalytic reaction. If no HRP is present on the modified electrode, the electrochemical reduction of TCA cannot be realized with no peaks appearing (curve b). The electrocatalytic mechanism was proposed as follows [52]:

HRP Fe(III) + H^+^ + e^−^ ↔ HRP Fe(II)

2[HRP Fe(II)] + Cl_3_CCOOH + H^+^ → 2[HRP Fe(III)] + Cl_2_CHCOOH + Cl^−^

Therefore, HRP-based Nafion/BPQDs/CILE was a typical electrocatalytic process towards TCA. The apparent Michaelis constant (*K_M_^app^*) is an important index of enzyme–substrate reaction kinetics. According to the Lineweaver–Burk equation [54], the *K_M_^app^* of the catalytic reaction could be calculated to be 0.46 mmol/L, which was smaller than that of the reported value [48,53]. The smaller the *K_M_^app^*, the better the affinity of the enzyme to the substrate, and the stronger the catalytic ability of the enzyme, which indicated that this modified electrode had higher electrocatalytic activity.

**Figure 7:**
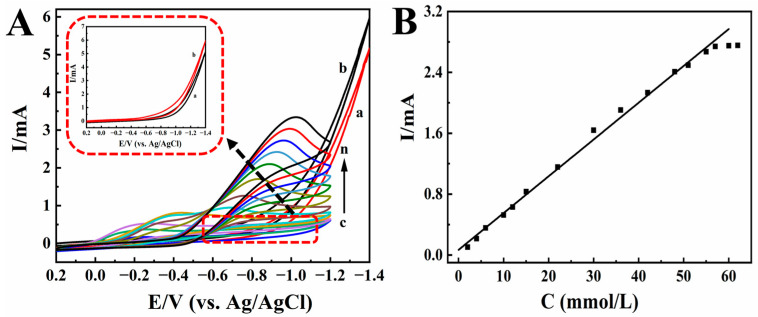
(**A**) Cyclic voltammograms of Nafion/BPQDs/CILE in 0.1 mol/L pH 2.0 PB with (a) 0.0, (b) 3.5 mmol/L KBrO_3_, and Nafion/HRP/BPQDs/CILE with 2.0, 4.0, 6.0, 10.0, 12.0, 15.0, 22.0, 30.0, 36.0, 42.0, 51.0, 57.0 mmol/L KBrO_3_ (curves c–n) at the scan rate of 100 mV/s. Inset was magnification of curves a and b. (**B**) Linear relationship of catalytic reduction peak currents and KBrO_3_ concentration.

KBrO_3_ is mainly used as an analytical reagent, oxidant, and food additive. Foods containing KBrO_3_ for a long time will cause great harm to the kidneys of the body. Therefore, it is very important to detect the content of KBrO_3_ in foods [55]. Figure 7A shows the electrocatalytic effect of Nafion/HRP/BPQDs/CILE on different concentrations of KBrO_3_. A new reduction peak appeared and moved negatively (curves c-n) with increasing concentration of KBrO_3_, and the reduction peak current also increased gradually. The electrocatalytic mechanism was proposed as follows [56]:

HRP Fe(III) + H^+^ + e^−^ → HRP Fe(II)

6HRP Fe(II) + BrO_3_^−^ + 6H^+^ → 6HRP Fe(III) + Br^−^ + 3H_2_O

When the concentration of KBrO_3_ was 2.0 to 57.0 mmol/L, there was a good linear relationship between the reduction peak current and the concentration of KBrO_3_ (Figure 7B). The linear regression equation was Ip (µA) = 0.0483 C (mmol/L) + 0.0681 (γ = 0.996), and the detection limit was 0.18 mmol/L (3S/N). According to the Lineweaver–Burk equation, the *K_M_^app^* was 0.052 mmol/L. Also, CV curves of Nafion/BPQDs/CILE with the addition of KBrO_3_ (curves a and b) were recorded, which did not exhibit any responses, indicating that the electro-reduction to KBrO_3_ had not taken place without HRP. The results also showed that this HRP-modified electrode had a good catalytic effect on KBrO_3_.

A systematic comparison with different modified electrodes for electrocatalytic detection of TCA and KBrO_3_ is listed in Table 1. This modified electrode indicated a relatively wider linear range and lower detection limit for the detection of the target analytes with a simple preparation procedure, which also extended the applications of BP-related nanomaterial in an electrochemical sensor.

### 2.4. Analytical Applications

To check the application to practical samples, Nafion/HRP/BPQDs/CILE was used to determine the content of TCA in medical facial peel solution and KBrO_3_ in flour supernatant by standard calibration. Furthermore, different concentrations of standard solutions were added to the sample solutions to calculate the recovery by the standard addition method using the same procedure. The contents of TCA and KBrO_3_ in the sample solutions were calculated as 11.20 mmol/L and 0 mmol/L, which were in accordance with the content of the real sample. The recovery was further calculated as 97.0% to 109.1% for TCA and 97.3% to 103.0% for KBrO_3_, with the results shown in Table 2. All the results proved that this method could be used for the analysis of TCA and KBrO_3_ in real samples with good applications.

### 2.5. Interference Test

**Figure 8:**
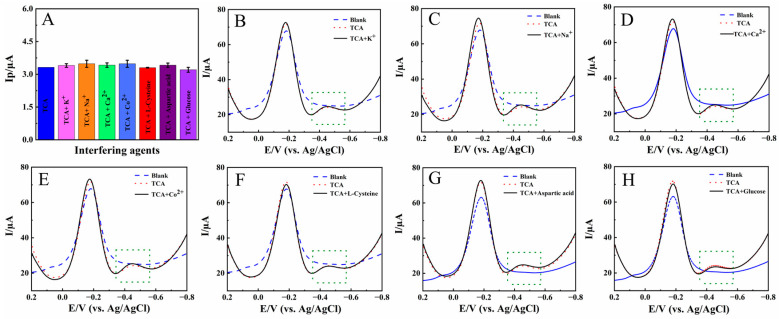
(**A**) The reduction peak currents of Nafion/HRP/BPQDs/CILE for the detection of 4.0 mmol/L TCA with the presence of different interfering agents in pH 2.0 PB (green dotted box); DPV curves of Nafion/HRP/BPQDs/CILE for the detection of 4.0 mmol/L TCA with the presence of 1.0 mmol/L different interfering agents: K^+^ (**B**), Na^+^ (**C**), Ca^2+^ (**D**), Co^2+^ (**E**), L-Cysteine (**F**), aspartic acid (**G**), glucose, (**H**) in pH 2.0 PB.

The anti-interference ability of Nafion/HRP/BPQDs/CILE was investigated in the presence of 1.0 mmol/L foreign agents, such as K^+^, Na^+^, Ca^2+^, Co^2+^, L-cysteine, aspartic acid, and glucose, in pH 2.0 PB, with 4.0 mmol/L TCA as the model. The results are shown in Figure 8A, with the original differential pulse voltammetry (DPV) curves presented in Figure 8B–H. It can be seen that the presence of some interfering analytes led to positive interference, with the response current increased. However, the relative error was less than 5.0% at the selected concentration, which was in the normal range of relative error and did not interfere with the target analysis. Therefore, Nafion/HRP/BPQDs/CILE had good anti-interference ability.

### 2.6. Stability and Repeatability of the Modified Electrode

**Figure 9:**
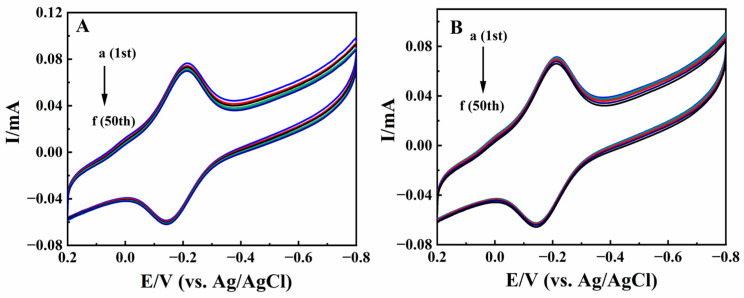
(**A**) Multi–scan cyclic voltammograms of Nafion/HRP/BPQDs/CILE in pH 2.0 PB with scan rate of 100 mV/s; (**B**) Multi–scan cyclic voltammograms of Nafion/HRP/BPQDs/CILE after storing 7 days with the scan rate of 100 mV/s (a to f represent 1, 10, 20, 30, 40, 50 circles).

The stability of Nafion/HRP/BPQDs/CILE was studied by multi-scan cyclic voltammetry, and the results are shown in Figure 9A. After 50 consecutive cycles of scanning, the current and potential of the redox peak of the modified electrode remained basically unchanged, and the deviation of the redox peaks current between the 1st lap and the 50th lap was 3.08% and 2.58%. It was proved that Nafion/HRP/BPQDs/CILE had good stability and reproducibility in N_2_-saturaterd PB. After the modified electrode was stored for 20 days at 4 °C, the cyclic voltammetric curve was measured again. After 50 cycles of continuous scanning, it was found that the redox peak current was retained at 94.7% and 92.3% of its original value, respectively (Figure 9B), which demonstrated that Nafion/HRP/BPQDs/CILE had good storage stability and a long lifetime. Five parallel modified electrodes were used to detect 20.0 mmol/L TCA with the RSD value of 3.65% and 10.0 mmol/L KBrO_3_ with the RSD value of 2.92%, demonstrating the good repeatability of Nafion/HRP/BPQDs/CILE.

## 3. Materials and Methods

### 3.1. Reagents and Chemicals

Ionic liquid 1-hexylpyridinium hexafluorophosphate (HPPF_6_, >99%, Lanzhou Yulu Fine Chem. Ltd., Co., Lanzhou, China), HRP (MW. 40000, Sinopharm Chem. Reagent Co., Shanghai, China), black phosphorus nanoplates dispersion (BPNPs, Nanjing XFNANO Materials Tech. Ltd., Co., Nanjing, China), trichloroacetic acid (TCA, Tianjin Kemiou Chem. Co., Tianjin, China), graphite powder (particle size 30 μm, Shanghai Colloid Chem. Co., Shanghai, Shanghai, China), potassium bromate (KBrO_3_, Shanghai Aladdin Regent Ltd., Co., Shanghai, China), potassium hexacyanoferrate (III) (K_3_[Fe(CN)_6_], Guangzhou Chem. Reagent Co., Guangzhou, China), Nafion ethanol solution (5.0%, Beijing Honghaitian Tech. Co., Beijing, China), and 1-methyl-2-pyrrolidinone (NMP, 99.5%, Shanghai Aladdin Regent Ltd., Co., Shanghai, China) were used directly.

The supporting electrolyte was 0.1 mol/L phosphate buffer (PB) solution with various pH values and deoxygenated by using pure nitrogen for 30 min before the experiments. All the other chemicals were of analytical grade, and ultra-pure water from a Mill-Q water purification system (Milli-Q IQ7000, Boston, MA, USA) was used throughout the experiments.

### 3.2. Apparatus

A CHI 1040C electrochemical workstation (Shanghai CH Instrument, Shanghai, China) was used for all the electrochemical experiments. A traditional three-electrode system was used, with the self-made modified electrode (Nafion/HRP/BPQDs/CILE) as the working electrode, a platinum wire as the auxiliary electrode, and an Ag/AgCl (saturated KCl) electrode as the reference electrode. Scanning electron microscopy (SEM) was performed on a JSM-7100F (JEOL Electron Co., Tokyo, Japan) with transmission electron microscopy (TEM) on a JEM-2010F (JEOL Electron Co., Tokyo, Japan). Ultraviolet-visible (UV–Vis) absorption spectra were performed on a UV-5 spectrophotometer (Mettler Toledo, Columbus, OH, USA), with Fourier-transform infrared spectroscopy (FT-IR) recorded on a Tensor 27 FT-IR spectrophotometer (Bruker Optics, Karlsruhe, Germany).

### 3.3. Preparation of BPQDs

Combining ultrasonic-assisted liquid-phase exfoliation and centrifugation could be used to obtain BPQDs, as described in the previous work [22]. Therefore, the general preparation procedure was described as follows. First, 5.0 mL 1.0 mg/mL BPNPs and 5.0 mL NMP were mixed and stirred, and sonicated in an ice bath for 8 h. The dispersion was centrifuged at 7000 rpm and 10,000 rpm for 20 min each to obtain BPQDs.

### 3.4. Preparation of BPQDs Modified Electrode

**Scheme 1:**
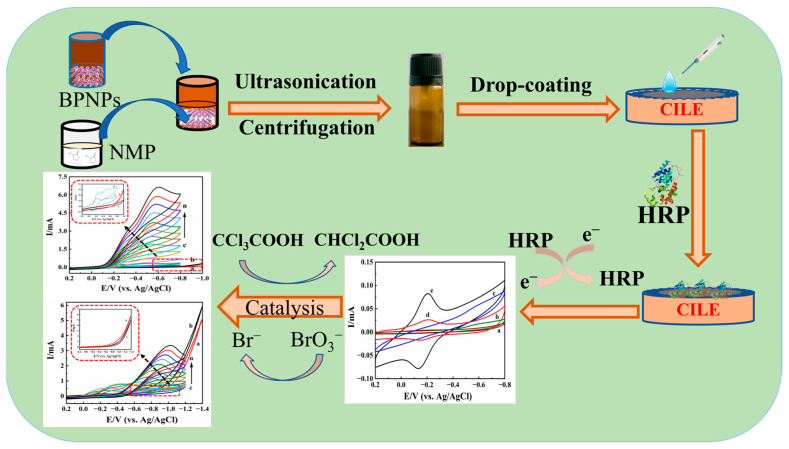
Fabrication procedure of Nafion/HRP/BPQDs/CILE.

CILE was constructed with graphite power and HPPF_6_ (mass ratio of 2:1), according to the previously reported procedure; it was used as the substrate electrode, and its surface was gently smoothed before use [67]. In a nitrogen-filled glove box, 10.0 μL of 0.5 mg/mL BPQDs solution was applied on the CILE surface and dried naturally to obtain the BPQDs/CILE, which was further coated by 10.0 μL of 15.0 mg/mL HRP solution and 10.0 μL of 0.5% Nafion ethanol solution in sequence with the whole process dried under nitrogen. Other electrodes were fabricated using the same procedure for comparison, and the preparation process of the working electrode is shown in Scheme 1.

### 3.5. Samples Analysis

Medical facial peel solution (35% TCA) was purchased from Shanghai EKEAR Bio. Tech. Co., (Shanghai, China) and used as the real sample for TCA analysis, which was directly diluted by the PB. The flour (purchased from Haikou Guilinyang farm product market) was selected as the sample for KBrO_3_ analysis. The sample solution was prepared by dispersing 10.0 g flour into 100 mL distilled water, sonicating for 30 min, and then centrifuging for 20 min at 10,000 rpm to obtain supernatant, which was used as the samples for further analysis.

## 4. Conclusions

In this paper, highly ambient-stable BPQDs were prepared by ultrasonic-assisted liquid-phase exfoliation and centrifugation using multilayer BPNPs. BPQDs were modified on the surface of CILE by layer-by-layer coating and used as the base electrode, then HRP was fixed on the BPQDs/CILE surface, and Nafion was used as a protective film to prepare a modified electrode (Nafion/HRP/BPQDs/CILE). A pair of well-shaped redox peaks appeared on cyclic voltammograms, and the electrocatalytic performances of the modified electrode were tested. The electrocatalytic behaviors of TCA and KBrO_3_ were investigated, and the wide linear detection ranges were 4.0–600.0 mmol/L and 2.0–57.0 mmol/L, with a low detection limit of 0.03 mmol/L and 0.18 mmol/L, respectively. Due to the specific characteristic of BPQDs, such as large specific surface areas, good electrical conductivity, and abundant active center, the direct electrochemistry of HRP was achieved with an enhanced redox peak current, which proved the positive effects of BPQDs on the electrode surface on the electron transfer of the HRP Fe(III)/Fe(II) redox center to exchange electrons with CILE. The excellent biocompatibility and high specific surface area of BPQDs proved that the constructed electrochemical HRP biosensor had an excellent electrochemical response and high electrocatalytic activity, and could be applied to the actual sample detection.

## Figures and Tables

**Table 1:** Comparison of analytical performances of different modified electrodes for TCA and KBrO_3_ analysis.

Modified Electrodes	TCA		Ref.	Modified Electrodes	KBrO_3_		Ref.
	Linear Range (mmol/L)	Detection Limit (mmol/L)			Linear Range (mmol/L)	Detection Limit (mmol/L)	
Nafion/Mb/TiO_2_@CNF/CILE	5.0–105.0	1.6	[52]	PdNPs/PANI/SBA-15/GCE	0.008–40.0	0.005	[31]
TH/TNTs/CS/GCE	0.015–1.5	/	[56]	Nafion/Hb/AuNPs/ND/CILE	0.01–12.0	0.0033	[55]
CTS/ELDH-GR-Hb/CILE	5.0–135.0	1.506	[57]	rGO-PDDA/PMo12/GCE	0.02–10.0	/	[58]
Hb/PDDA/PGE	3.92–58.4	1.98	[59]	Cd-IL/CPE	5.0–20.0	3.0	[60]
Nafion/Hb/GQD/CILE	6.0–100.0	2.0	[61]	MWCNT/Pd/GCE	1.0–40.0	/	[62]
np-Ag electrode	2.5–22.5	0.25	[63]	Tungsten oxide electrode	0.3–45.0	0.1	[64]
CdOMCPE	0.23–0.003	0.0023	[65]	Fe(III)P/MWCNTs/GCE	0.5–3.5	/	[66]
Nafion/HRP/BPQDs/CILE	4.0–600.0	0.03	This work	Nafion/HRP/BPQDs/CILE	2.0–57.0	0.18	This work

Notes: CNF: carbon nanofiber; TH: thionine; TNTs: titanate nanotubes; CS: chitosan; CTS: chitosan; ELDH: exfoliated Co_2_Al layered double hydroxide; GR: graphene; PDDA: Poly(diallyldimethylammonium); GQD: graphene quantum dots; np-Ag: nanoporous silver; CDOMCPE: CdO modified carbon paste electrode; PANI: polyaniline; SBA-15: mesoporous; ND: nano-diamond; rGO: graphene oxide; PMo12: Phosphomolybdate; MWCNT: multiwall carbon nanotubes; Fe(III)P: 5,10,15,20-Tetraphenyl-21H,23H-porphine iron(III) chloride.

**Table 2:** Analytical results of real samples using this electrochemical sensor (*n* = 3).

Samples	Added (mmol/L)	Total (mmol/L)	Recovery (%)	RSD (%)
Medical facial peel solution	10.00	20.90	97.0	3.21
	20.00	33.01	109.1	3.67
	30.00	43.54	107.8	2.81
Flour supernatant	2.00	1.98	99.0	2.13
	4.00	4.12	103.0	3.36
	6.00	5.84	97.3	2.91

## Data Availability

Not applicable.

## Sample Availability

Samples of the compounds are available from the authors.
